# Late-onset obsessive–compulsive phenomenology with parkinsonian features: a neuropsychiatric interface case

**DOI:** 10.1192/j.eurpsy.2026.11604

**Published:** 2026-07-31

**Authors:** L. M. M. C. Cardoso Carrasqueira

**Affiliations:** Serviço de Psiquiatria e Saúde Mental, Unidade Local de Saúde da Região de Leiria, Leiria, Portugal

## Abstract

**Introduction:**

Late-onset obsessive-compulsive symptoms are less common than early-onset forms and can indicate disruptions in fronto-subcortical circuits from neurodegenerative,vascular or other organic etiologies. This case study highlights the intersection of obsessive-compulsive phenomenology and parkinsonian features, focusing on a patient with subtle motor signs.

**Objectives:**

The primary objective of this case study is to elucidate the diagnostic and clinical challenges encountered in managing late-onset obsessive-compulsive symptoms with parkinsonian features, emphasizing the importance of suspecting an underlying cause.

**Methods:**

The case study was developed through comprehensive psychiatric and neurological assessments during the patient’s outpatient follow-up, supplemented by a targeted literature review on PubMed using “late-onset obsessive-compulsive disorder” and “parkinsonian features” as keywords.

**Results:**

The patient, a 60-year-old male, was a functional adult with chronic anxiety and subclinical obsessive-compulsive traits until approximately two years prior when he began exhibiting symptoms of late-onset obsessive-compulsive phenomenology. His symptoms progressively evolved from intrusive catastrophic thoughts to rigid, stereotyped motor rituals impacting daily activities (e.g., dressing, gait, feeding), interpreted as corrections for bodily misalignment. As the condition worsened, insight was partially preserved but resistance waned, leading to significant functional impairment without classic obsessive-compulsive disorder (OCD) forms like washing or checking, and with comorbid depression. Neurological examination revealed hypomimia, psychomotor slowing, and subtle parkinsonian gait without rigidity; Mini-Mental State Examination (MMSE) scored 30/30. Cranial computed tomography (CT) showed frontal-predominant microangiopathic leukoencephalopathy, and due to suspicion of underlying pathology, magnetic resonance imaging (MRI) confirmed vascular changes without additional findings. Prior treatments encompassed standard selective serotonin reuptake inhibitors (SSRIs), venlafaxine, clomipramine, and atypical antipsychotics; current management involves high-dose fluvoxamine with low-dose antipsychotic augmentation and frequent evaluations in ambulatory.

**Conclusions:**

This case illustrates the importance of integrated neuropsychiatric assessment with longitudinal monitoring in patients presenting late-onset obsessive-compulsive symptoms. The presence of stereotyped motor rituals emerging in adulthood with parkinsonian features warrants screening for neurodegenerative or vascular pathology rather than assuming primary obsessive-compulsive disorder. This multifaceted approach is crucial in addressing the complex needs of patients with late-onset obsessive-compulsive phenomenology at the neuropsychiatric interface.

**Disclosure of Interest:**

None Declared

